# Editorial: Vol II: person-centred rehabilitation - theory, practice and research

**DOI:** 10.3389/fresc.2025.1665527

**Published:** 2025-07-29

**Authors:** Jacqui H. Morris, Nicola Kayes, Lesley Scobbie, Brendan McCormack

**Affiliations:** ^1^ School of Health Sciences, University of Dundee, Dundee, United Kingdom; ^2^ Centre for Person Centred Research, Aukland University of Technology, Aukland, New Zealand; ^3^ Department of Occupational Therapy, Human Nutrition and Dietetics, Glasgow Caledonian University, Glasgow, United Kingdom; ^4^ The Susan Wakil School of Nursing and Midwifery, University of Sydney, Sydney, NSW, Australia

**Keywords:** rehabilitation, person-centred, personhood, patient care, philosophy

**Editorial on the Research Topic**
Vol II: person-centred rehabilitation – theory, practice and research

The concept of person-centredness is widely advocated in health policy, service frameworks and clinical practice (1). It is positioned as a philosophical approach to healthcare based on humanistic principles, including dignity, self-actualisation, empathy and genuine care relationships (2, 3). Person-centredness requires the development of collaborative relationships between persons delivering and receiving care that foster interconnectedness, interdependence and shared responsibilities (4). Person-centredness seeks to facilitate autonomy and empowerment of and shared decision-making with those receiving care. It recognises and treats individuals as social and relational beings influenced by multi-factorial environments, psychological, social and biographical histories and experiences (2, 4, 5).

Person-centredness in care is derived from a philosophical stance of personhood - that individuals have the characteristics, attributes and status of a person and morally should be treated as such irrespective of their capacities or capabilities (2). Additionally, person-centred approaches in healthcare also recognise the relevance of the related concept of selfhood (3, 4, 6). Simply put, selfhood is understood as one's personal or self-identity defined in social and relational contexts and is recognised to evolve and change over time and in response to life events (7). Selfhood is layered, fluid, responsive and autobiographical and is shaped by interactions, relationships, experiences, roles and events, rather than capabilities at a moment in time. While personhood and selfhood are distinct concepts, they are also related. Selfhood can be seen as a component of being a person through a person's subjective articulation of what defines them as a unique individual.

People receiving rehabilitation have often experienced illness or injury, or chronic and progressive conditions and impairments that disrupt their life trajectory and sense of self in terms of physical, emotional, social and occupational functioning. This impact of illness on individuals’ life narrative has long been described as biographical disruption, where challenges threaten previous assumptions about the future and one's self-identity or selfhood (8). The effect of biographical disruption on individuals’ sense of self is encapsulated by the often-quoted statement “I don't know who I am any more” (9). It is the impact of illness or injury beyond the physical or functional and the related altered subjective perspective on personhood that make the notion of selfhood highly relevant to rehabilitation (9).

Despite this, and the espousal of the biopsychosocial model of rehabilitation that underpins training and practice for rehabilitation professionals, the traditional basis for rehabilitation practice that stemmed from a biomedical perspective still prevails (1, 10). Rehabilitation has often been criticised for its focus on physical and functional recovery, at the expense of recognising the profound social and psychological impact that illness can have on a person, and the compound effects that these disruptions have on selfhood (9).

Despite the tendency for biomedical perspectives to dominate, there is a growing body of work around person-centred rehabilitation research. This work informs the development of models and frameworks that conceptualise person-centred rehabilitation and develop and test rehabilitation interventions and processes for practice and service delivery (1, 10). The shift towards person-centred rehabilitation is reflected in the papers included in this research topic, which span conceptualisation and reporting of illness experiences to inform person-centred rehabilitation; training of rehabilitation professionals in person-centred goal setting; and case study examples of person-centred practice.

Two papers illustrate the importance of time and temporality on influences on selfhood in rehabilitation. In their qualitative paper investigating the reconfiguration of identity after stroke Hall et al. explored the narratives of 30 people with stroke, exploring individual and social factors that influence reconfigurement of post-stroke self-identity over time. Using grounded theory, the authors developed a model based on the concept of liminality, the idea that identity undergoes reconfigurations as people with stroke experience attendant uncertainties in their new situation. The model reflects and conceptualises the ongoing transitional states experienced between pre and post-stroke selfhood, providing novel insights into the experiences of stroke and stroke rehabilitation. It also provides a novel platform that could be applied in other conditions.

Similarly, in a qualitative literature synthesis Njest and Glintborg examined the concept of hope in rehabilitation after traumatic brain injury, finding that it is a multi-faceted and complex construct for people receiving and delivering rehabilitation. Integrally linked with goals for the future, hope could be a negative influence – encapsulating despair and hopelessness; as well as a positive driver for future progress. As with liminality, hope is integrally linked to temporality, with ongoing uncertainty underpinning the sense of movement between past and future identities and sense of selfhood. These conceptually complex ideas encapsulate the often-unrecognised struggles experienced by people receiving rehabilitation. They also emphasise the need for rehabilitation professionals to be aware of their own sense of self and personhood (5) to be able to support the people they work with to move beyond simple physical and functional goals.

Enjoyment is another often neglected dimension of rehabilitation and is here examined by Dahl et al. Exploring the experiences of people with multiple sclerosis participating in high-intensity impact training, the authors found that creating an engaging environment with enjoyment as a key characteristic led to positive changes for most in motivation, perceptions of physical capability, and confidence. Crucially, the co-creation of enjoyment by participants was a key influencing factor on how people experienced the training and provides a key lesson for those designing rehabilitation activities and interventions in person-centred ways. These findings resonate with theory and evidence regarding the value and effectiveness of autonomy-supportive rehabilitation strategies and environments (11).

Two case studies provide examples of rehabilitation delivered in person-centred ways. In the study by Brennan et al. aural rehabilitation was delivered to a professional musician whose hearing loss and subsequent use of a cochlear implant and hearing aids meant that music perception was distorted. The loss of musical enjoyment profoundly influenced the musician's perceived selfhood as a professional musician. Working with her to re-establish musical enjoyment through selection of previously valued musical pieces is a clear example of how being attuned to what is personally meaningful and tailoring rehabilitation to what matters to people can contribute to restoring one's sense of self-identity. Similarly, in a case study with a person experiencing dysarthria resulting from supranuclear palsy, Sebestyen et al. report how vocal rehabilitation improved and sustained vocal quality and intelligibility. Being able to be heard by others, be understood and able to participate in social interactions is a crucial facet of personhood that influences sense of identity (4). This example illustrates the impact person-centred rehabilitation practice can have on sense of self. Both examples show how biographical work can be integrated into the rehabilitation process.

Shared, meaningful goal setting is a key vehicle for communication and delivery of person-centred practice in rehabilitation and is known to enhance recipients’ confidence, engagement and motivation and satisfaction, enabling them to engage in and achieve outcomes that are personally meaningful (12). However, there is a wealth of literature suggesting that goal setting is often led by professionals and addresses their priorities, rather than those of the people they work with (12). Training to enhance rehabilitation professionals’ understanding and implementation of person-centred goal setting practice is therefore vital if we are to move towards authentic person-centred rehabilitation. Scobbie et al. have developed an evidence and theory-based Goal-Setting and Action Planning (G-AP) framework intended to support the setting and pursuit of person-centred goals in rehabilitation. In this paper they describe the development and evaluation of an online and webinar-based training resource to support rehabilitation practitioners to implement G-AP in practice. They demonstrated the success of training in increasing knowledge and confidence in use of G-AP. Whilst individuals were able to implement G-AP into practice, there were however challenges in implementing it at team level, highlighting the complexity of contextual factors, including time, willingness and staff capacity in developing a culture in which person-centredness is the norm.

This second volume provides examples of research illustrating the complex theoretical and practice-related landscape of person-centred rehabilitation and demonstrates how person-centredness in rehabilitation is advancing. In some regards, rehabilitation research and practice is in a golden era. The last two decades have seen an explosion in rehabilitation research, in the development and testing of new interventions and in the conceptualisation of rehabilitation and rehabilitation practice. Reflecting global recognition of the importance of rehabilitation, the WHO has asserted that rehabilitation is a human right and has positioned rehabilitation provision as a global priority (13). However, as this volume illustrates, intervention development and testing, implementation and philosophical, theoretical and practice-related person-centred constructs and ideas do not exist in a vacuum. Of equal importance is the understanding and development of cultures, contexts and environments in which person-centred rehabilitation can be the norm. There have been important strides in this direction. Kayes and Papadimitriou (1) illustrate the challenges to person-centred rehabilitation at the levels of concept and practice but also present a model of person-centred culture in rehabilitation that illustrates the macro environments that have to change to facilitate and sustain person-centred rehabilitation.

The recently published model of Person-Centred Rehabilitation (10) encapsulates in detail the multidimensional components of macro-system (organisational level), micro-system (rehabilitation team level) and person-professional dyad level required to support person-centred rehabilitation. Clearly, there is a moral imperative for thinkers and policy, research and service leaders in rehabilitation, and practitioners, to not only espouse person-centred policies and practice but to go beyond that. As this volume illustrates, we require theoretical and conceptual understanding of person-centred rehabilitation, and evidence-based strategies to embed person-centredness in healthcare practice, culture and services. Only in this way will we support people receiving rehabilitation to re-establish meaningful selfhood that goes beyond physical and functional outcomes.
